# Activation and Inhibition of Transglutaminase 2 in Mice

**DOI:** 10.1371/journal.pone.0030642

**Published:** 2012-02-02

**Authors:** Laila Dafik, Megan Albertelli, Jorunn Stamnaes, Ludvig M. Sollid, Chaitan Khosla

**Affiliations:** 1 Department of Chemistry and Chemical Engineering, Stanford University, Stanford, California, United States of America; 2 Department of Comparative Medicine, Stanford University, Stanford, California, United States of America; 3 Centre for Immune Regulation and Department of Immunology, University of Oslo, Oslo, Norway; 4 Oslo University Hospital- Rikshospitalet, Oslo, Norway; 5 Department of Biochemistry, Stanford University, Stanford, California, United States of America; University of Nebraska Medical Center, United States of America

## Abstract

Transglutaminase 2 (TG2) is an allosterically regulated enzyme with transamidating, deamidating and cell signaling activities. It is thought to catalyze sequence-specific deamidation of dietary gluten peptides in the small intestines of celiac disease patients. Because this modification has profound consequences for disease pathogenesis, there is considerable interest in the design of small molecule TG2 inhibitors. Although many classes of TG2 inhibitors have been reported, thus far an animal model for screening them to identify promising celiac drug candidates has remained elusive. Using intraperitoneal administration of the toll-like receptor 3 (TLR3) ligand, polyinosinic-polycytidylic acid (poly(I∶C)), we induced rapid TG2 activation in the mouse small intestine. Dose dependence was observed in the activation of TG2 as well as the associated villous atrophy, gross clinical response, and rise in serum concentration of the IL-15/IL-15R complex. TG2 activity was most pronounced in the upper small intestine. No evidence of TG2 activation was observed in the lung mucosa, nor were TLR7/8 ligands able to elicit an analogous response. Introduction of ERW1041E, a small molecule TG2 inhibitor, in this mouse model resulted in TG2 inhibition in the small intestine. TG2 inhibition had no effect on villous atrophy, suggesting that activation of this enzyme is a consequence, rather than a cause, of poly(I∶C) induced enteropathy. Consistent with this finding, administration of poly(I∶C) to TG2 knockout mice also induced villous atrophy. Our findings pave the way for pharmacological evaluation of small molecule TG2 inhibitors as drug candidates for celiac disease.

## Introduction

Transglutaminase 2 (TG2, a.k.a. tissue transglutaminase) is a ubiquitous multifunctional mammalian protein that catalyzes the formation of intermolecular isopeptide bonds between glutamine and lysine residues of selected proteins [1]–[3]. Its enzymatic activity is allosterically regulated by several factors, including guanine nucleotides, Ca^+2^, and redox potential [4]–[6]. In pathological situations, such as in the small intestinal mucosa of celiac disease patients, TG2 can also deamidate glutamine residues of gluten peptides, creating potent T cell epitopes [7]–[9]. Therefore, TG2 inhibitors are thought to represent promising avenues for celiac disease therapy [9].

Although numerous small molecule TG2 inhibitors have been reported to date [10]–[16], an assay to compare their relative efficacy *in vivo* has remained elusive. The target organ for celiac disease therapy is the upper small intestine; however, TG2 is in a catalytically inactive state in the intestinal mucosa of healthy rodents [17]. Therefore, a prerequisite for assessing inhibitor pharmacodynamics is the development of a model system in which TG2 is activated in the upper small intestine in response to an inflammatory trigger. Recently, we reported that intraperitoneal injection of polyinosinic-polysytidylic acid (poly(I∶C)), a toll-like receptor 3 (TLR3) ligand, led to rapid activation of TG2 in the small intestinal mucosa of C57BL/6J mice [17]. Poly(I∶C) is a synthetic analog of double-stranded RNA that has been widely used to mimic viral infection. Our protocol, which was based on earlier reports demonstrating an enteropathic response to poly(I∶C) in mice [18], [19], set the stage for developing a pharmacological assay to measure the potency of small molecule TG2 inhibitors in the upper intestine. Here we characterize this assay in greater detail, and exploit it to identify a bona fide lead compound, ERW1041E, for celiac drug discovery.

## Results

### Dose dependence of the poly(I∶C) mediated inflammatory response

Earlier studies have shown that intraperitoneal injection of a single 30 mg/kg dose of poly(I∶C) in C57BL/6J mice induced severe small intestinal injury that is characterized by villous atrophy, an increase in serum concentrations of IL-15, and activation of TG2 [17], [18]. Activation of TG2, as measured by incorporation of the TG2 substrate 5-biotinylamide pentylamine (5BP), occurred within a few hours after poly(I∶C) administration, and was most pronounced at the villus tips. To explore the dose dependence of this acute inflammatory condition, we first sought to standardize the procedure for preparing poly(I∶C), because preliminary studies revealed that commercial poly(I∶C) was unsuitable for quantitative experimentation (data not shown).

**Figure 1:**
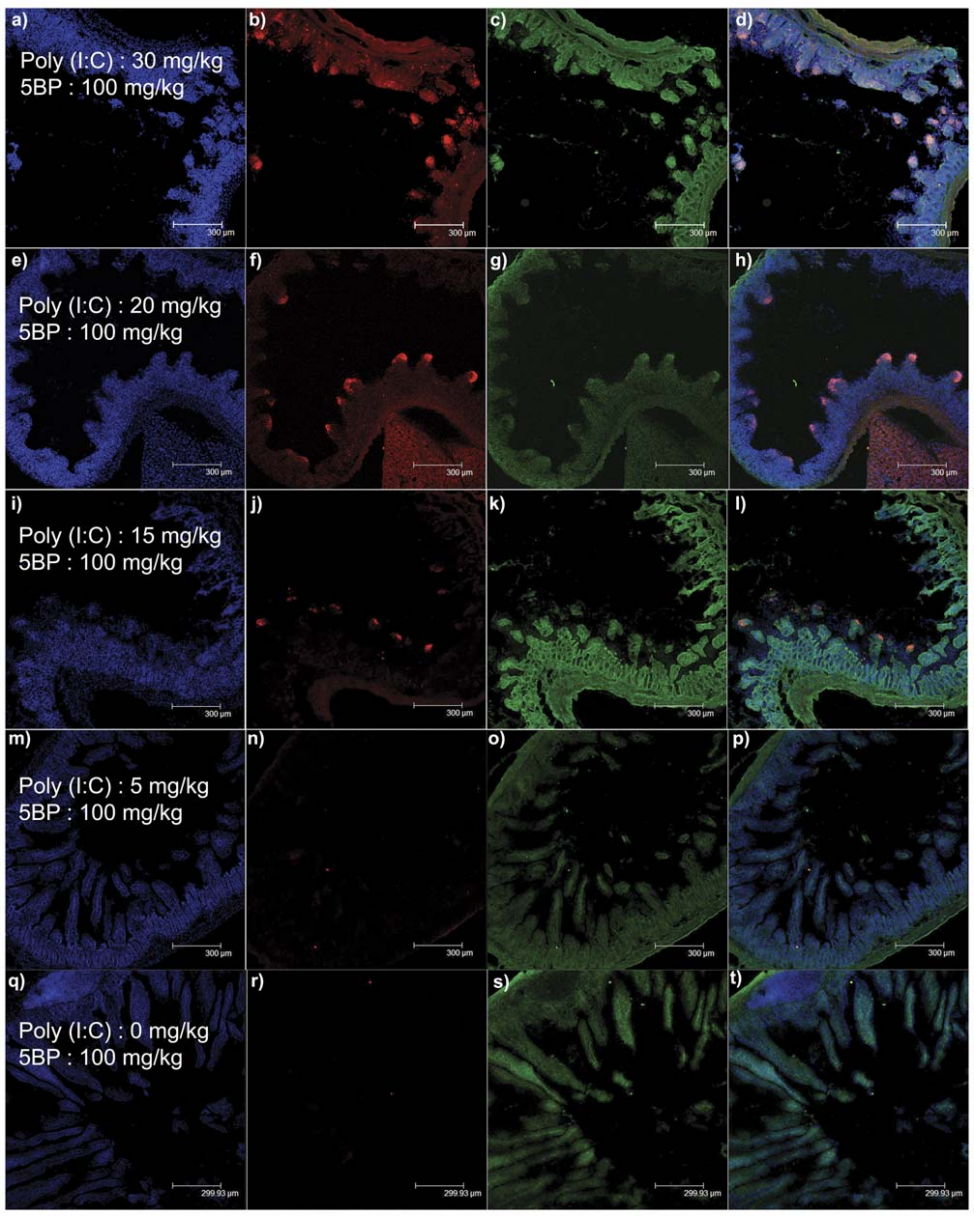
Treatment of C57BL/6J mice with poly(I∶C) results in villous atrophy and activation of TG2 at the villus tips. Single intraperitoneal injections of 30 (a–d), 20 (e–h), 15 (i–l), 5 (m–p), or 0 (q–t) mg/kg poly(I∶C) were administered. Mice were sacrificed after 9 h, and small intestines collected and frozen in OCT. Sections of 20 µm thickness were cut, fixed, washed, permeabilized, and exposed first to a rabbit anti-TG2 antibody (ab421) followed by a secondary Alexa fluor 488-congugated goat anti-rabbit IgG (c, g, k, o, s). Total TG2 protein (active and non active) is shown. TG2 activity after poly(I∶C) injury, measured using 5BP crosslinking, was visualized by co-staining with Alexa fluor 555-conjugated streptavidin (b, f, j, n, r). Nuclei were stained with DAPI (a, e, i, m, q). Overlays of the three stains are shown in (d, h, l, p, t). At least 3 animals were tested at each poly(I∶C) dose shown; one representative duodenal section is shown.

**Figure 2:**
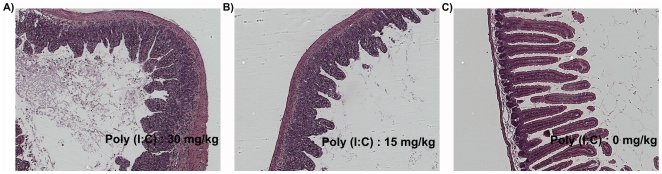
H&E staining of duodenal cross sections from mice treated with poly(I∶C) at 30 mg/kg (A), 15 mg/kg (B), or 0 mg/kg poly(I∶C) (C). Animals were sacrificed 9 h after poly(I∶C) administration, and their small intestines were harvested.

**Figure 3:**
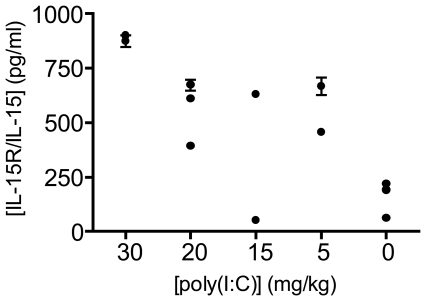
Serum levels of IL-15R/IL-15 in response to poly(I∶C). Treatment with poly(I∶C) increased the levels of IL-15R/IL-15 in serum. All of the mice in this experiment except those receiving no poly(I∶C) and one of the mice injected with 15 mg/kg poly(I∶C) (the one with low levels of IL15Rα/IL-15), developed enteropathy. Given values are mean of duplicate measurements for each mouse (error bars indicate SD for each measurement). The overall experiment was repeated twice with similar results.

Poly(I∶C) was dissolved in sterile PBS at room temperature. The solution was heated to 85°C for 3 min, and subsequently annealed by allowing it to cool by 1°C per min, until it reached room temperature. We have found that poly(I∶C) prepared by this procedure results in reproducible intestinal injury as compared to using it directly as purchased from the vendor. The final poly(I∶C) concentration was measured at 260 nm, and used to inject mice at 30, 20, 15, or 5 mg/kg. The duodenal mucosa of most mice exposed to the three highest doses revealed TG2 activation, especially at villus tips, with a clear dose-dependent pattern (Figure 1). Corresponding levels of villous atrophy were confirmed by H&E staining (Figure 2). Low levels of TG2 activity could also be detected in some mice injected with 5 mg/kg poly(I∶C) (Figure 1). Importantly, mice treated with 30 mg/kg showed severe acute symptoms and intestinal lesions, whereas lower poly(I∶C) doses did not elicit comparable effects. Intestinal sections collected from control cohorts treated with 0 mg/kg poly(I∶C) followed by 5BP showed normal histology with no TG2 activity (data not shown). The serum concentrations of the IL-15/IL-15R complex correlated well with histological and clinical severity of the animals (Figure 3). Both intestinal inflammation and TG2 activity were transient phenomena as mice treated with sub-lethal doses of poly(I∶C) recovered in 24–48 h (data not shown).

**Figure 4:**
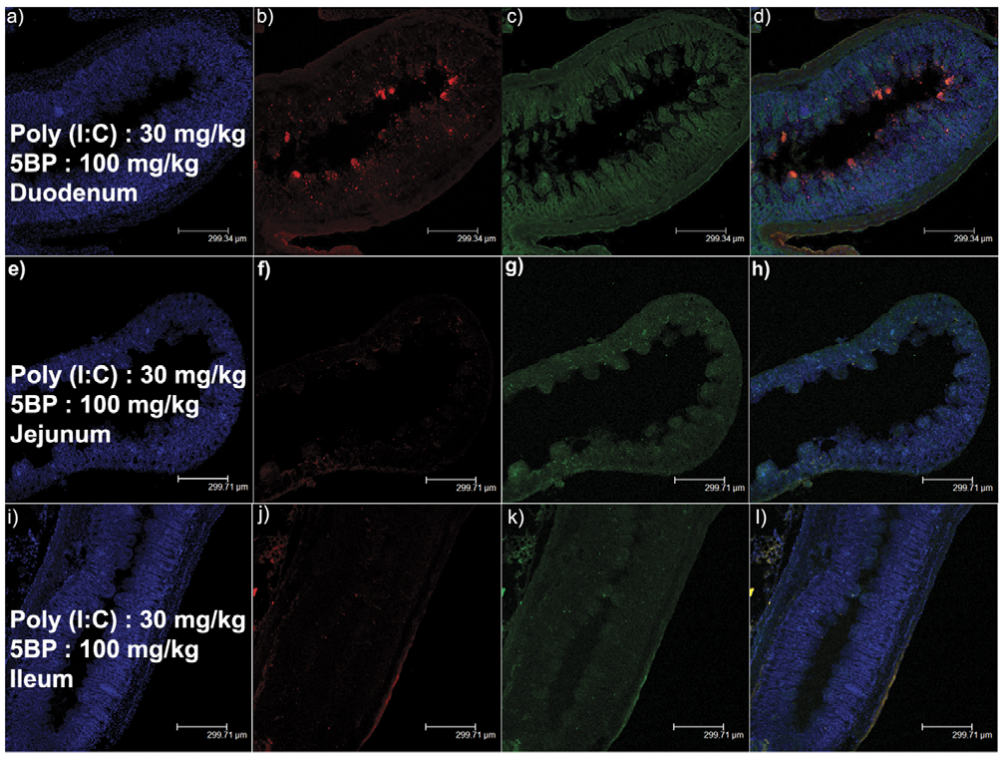
Localization of TG2 activity in the small intestine of poly(I∶C)-treated mice. Mice were treated with 30 mg/kg poly(I∶C), and samples were collected from the duodenum (a–d), jejunum (e–h), and ileum (i–l). Active TG2 was detected by exposing cross-sections to Alexa fluor 555-conjugated (b, f, j). The total amount of TG2 protein in these sections was visualized by co-staining with polyclonal anti-TG2 antibody, followed by an Alexa fluor 488-conjugated secondary antibody (c, g, k). Nuclei were stained with DAPI (a, e, i). Overlays are shown in the right column (d, h, l). Scale bar represents 300 µm and applies to all panels. At least 4 animals were tested; one representative section is shown for each intestinal segment.

To evaluate the tissue- and organ-specificity of poly(I∶C)-mediated TG2 activation, mucosa along the length of the intestine as well as the lungs were examined. As shown in Figure 4, TG2 activity was most pronounced in the duodenum, and decreased progressively in the jejunum and ileum. No evidence of 5BP crosslinking was observed in lung mucosa (data not shown). To determine whether other TLR ligands could induce TG2 activity in the small intestine, C57BL/6J mice were injected with R848, a potent TLR7/8 agonist [20]. Again, no changes were observed in either the gross histological architecture or the TG2 activity in the duodenum (data not shown).

### Inhibition of catalytically active TG2 in the small intestine by small molecule inhibitors

**Figure 5:**
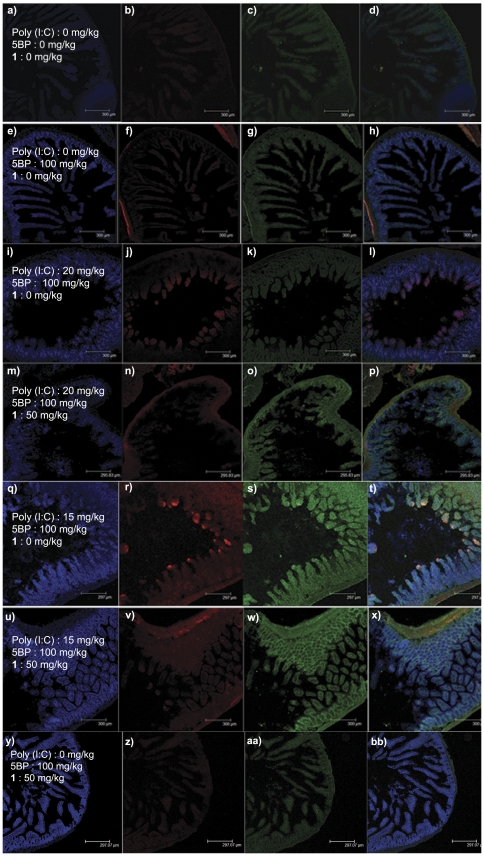
Inhibition of TG2 activity by ERW1041E in the small intestine of poly(I∶C)-treated mice. Mice were treated with 20 mg/kg (i–p), or 15 mg/kg (q–x) poly(I∶C), followed by two intraperitoneal injections of vehicle (i–l; q–t) or 50 mg/kg ERW1041E (m–p; u-bb) at 6 h and 9 h. Control cohorts were dosed with no pharmacological agent (a–d), only 5BP (e–h) or ERW1041E and 5BP (y-bb). TG2 activity was detected by exposing duodenal cross-sections to Alexa fluor 555-conjugated streptavidin (b, f, j, n, r, v, z). TG2 protein was visualized by co-staining with polyclonal anti-TG2 antibody, followed by a secondary antibody conjugated to Alexa fluor 488 (c, g, k, o, s, w, aa). Nuclei were visualized by staining with DAPI (a, e, i, m, q, u, y). The right column contains overlay images (d, h, l, p, t, x, bb). At least 3 animals were tested in each cohort; one representative duodenal section is shown.

Having characterized the dose-response of the mouse small intestine to poly(I∶C), we sought to determine whether transiently activated TG2 could be pharmacologically inhibited. To simulate sub-lethal inflammatory conditions, poly(I∶C) doses of 15 and 20 mg/kg were selected for these experiments. Over the course of these studies, we screened several TG2 inhibitors for efficacy and tolerability. One compound in particular, ERW1041E, was well tolerated in chronic administration regimens up to 50 mg/kg (Figure 5), and was also able to inhibit small intestinal TG2 activity in poly(I∶C) treated mice in a dose-dependent manner (data not shown). TG2 inhibition had no effect on the observed villous atrophy (data not shown), suggesting that activation of this enzyme is a consequence, rather than a cause, of poly(I∶C) induced enteropathy.

### Effect of poly(I∶C) in TG2 knockout mice

**Figure 6:**
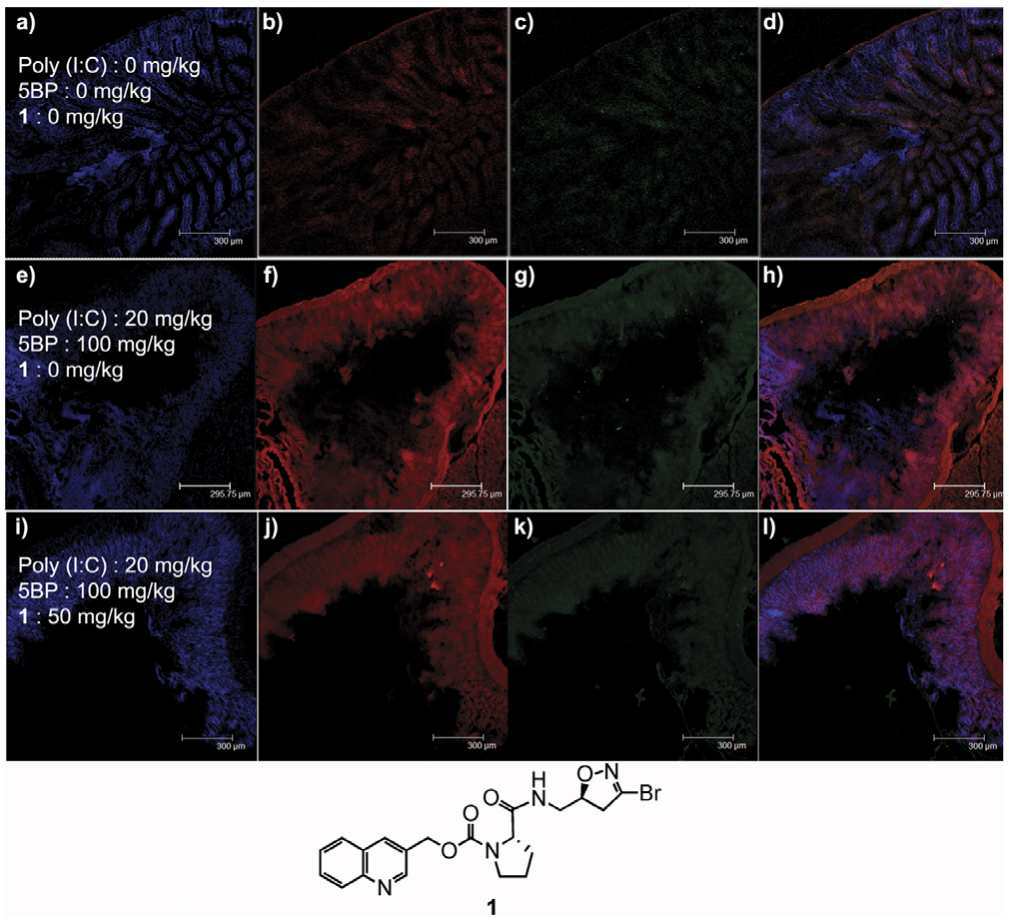
Effects of poly(I∶C) treatment on TG2 knockout mice. Mice were treated with 0 mg/kg (a–d) or 20 mg/kg (e–l) poly(I∶C), followed by two intraperitoneal injections of PBS (a–d), or 100 mg/kg (e–l) 5BP at 6 h and 9 h. Poly(I∶C)-treated mice were subsequently administered 50 mg/kg ERW1041E. TG2 activity was detected by exposing duodenal cross-sections to Alexa fluor 555-conjugated streptavidin (b, f, j). TG2 protein was visualized by co-staining with polyclonal anti-TG2 antibody, followed by a secondary antibody conjugated with Alexa fluor 488 (c, g, k). Nuclei were stained with DAPI (a, e, i). Overlays are shown in the right column (d, h, l). At least 3 animals were tested in each cohort; one representative duodenal section is shown. Unlike wild-type mice, significant lethality (2/7) was observed in poly(I∶C)-treated mice. The structure of ERW1041E (**1**) is shown.

To confirm that the elevated 5BP incorporation at duodenal villus tips was indeed due to TG2 activity, we examined the effect of poly(I∶C) in TG2-knockout mice [21]. Intraperitoneal injection of a single 20 mg/kg dose of poly(I∶C) induced small intestinal injury and villous atrophy (Figure 6), verifying that the inflammatory effects of poly(I∶C) did not depend upon TG2 activity. Indeed, the toxicity of poly(I∶C) appeared to be considerably more severe in TG2-knockout mice than in wild-type mice, as judged by the high lethality at the 20 mg/kg dose in the former strain (significant lethality (2/7) was observed in poly(I∶C)-treated mice). An intense distribution of 5BP was observed in duodenal cross-sections of TG2-knockout mice treated with poly(I∶C) (Figure 6f). However, in contrast to wild-type animals (e.g. see Figures 1f or 5j), the 5BP was uniformly distributed throughout the mucosa, and was not concentrated at the tips of blunted villi. Moreover, co-treatment of TG2-knockout mice with poly(I∶C) and the TG2 inhibitor ERW1041E showed no difference in the intensity or distribution of 5BP incorporation (Figure 6, panel f versus j). Together, these results demonstrate that TG2 is rapidly activated in duodenal villus tips of wild-type (but not TG2-knockout) mice, and that transient TG2 activity can be inhibited by small molecule inhibitors of this enzyme.

## Discussion

The search for TG2 inhibitors that are appropriate drug candidates for celiac disease therapy requires a number of successive pharmacological assays of increasing complexity and information content. Naturally, the primary screen is a kinetic assay for enzyme inhibition. Quantitative biochemical assays for TG2 activity are well established [22], [23], and can be implemented in a variety of moderately high-throughput formats for screening small molecules to identify TG2 inhibitors. As a next step, a cell-based assay is desirable to assess the tolerability and activity of promising inhibitors in biological systems. Because both intracellular and extracellular TG2 pools associated with typical cultured cell lines are catalytically inactive, we previously developed a fibroblast scratch assay for this purpose [17], [24]. TG2 is rapidly activated when WI-38 fibroblast cells grown on fibronectin-coated surfaces are gently scratched with a pipet tip, a phenomenon that mimics the well-documented role of TG2 in wound healing [17]. Activated TG2 can be either directly visualized with “clickable” active site-directed probes [24], or indirectly using the biotinylated amine 5BP, which crosslinks onto glutamine residues on accessible TG2 substrates [2]. For example, the minimum inhibitory concentration (MIC) of the dihydroisoxazole TG2 inhibitor, ERW1041E (**1**, [15], [24]) is 6.25–12.5 µM in this assay. In this study, we elicit small intestinal injury in C57BL/6J mice using poly(I∶C), and test the ability of ERW1041E to inhibit transiently activated TG2 in intestinal mucosa. To our knowledge, the current report is the first description of an *in vivo* assay for TG2 inhibition in the mammalian intestine.

Toll-like receptors (TLR) are innate immune system sensors that detect pathogen-associated molecules such as bacterial lipopolysaccharides and viral double-stranded RNA, and trigger a broader immune response. Poly(I∶C) is a TLR3 ligand that mimics viral infection. Treatment of mice with this pro-inflammatory agent induces villous atrophy in the small intestine, and also results in the activation of mucosal TG2. This response is localized to the small intestine, in particular to the duodenum and jejunum, and does not appear to be achievable by alternative TLR receptors. Thus, future efforts to develop non-dietary celiac therapies that block TG2 should be tested in this *in vivo* assay with ERW1041E as a reference compound.

Notwithstanding the pharmacological promise of this animal model, the assay has certain fundamental and practical limitations. First, the mechanism of inducing TG2 activity in the small intestine does not involve gluten, and is therefore not directly relevant to celiac disease pathogenesis. Although encouraging progress has been made recently towards the development of a mouse model for celiac disease [25], much remains to be learned about the mechanism by which gluten peptides induce TG2 activation in the celiac intestinal mucosa. It is likely that improved models for intestinal TG2 activation and inhibitor evaluation will emerge, as more insight emerges into this phenomenon of critical importance to celiac disease pathogenesis. Second, whereas a fundamental premise of TG2 blockers as celiac drug candidates is that inhibition of this target can prevent intestinal inflammation in a patient, our results clearly demonstrate that TG2 activity is not a requisite for poly(I∶C) mediated intestinal inflammation. Thus, even though our animal model can facilitate quantitative discrimination between alternative drug candidates, it does not test the core hypothesis underlying this mode of action of a celiac drug candidate. In this regard it may be worth assessing intestinal TG2 activity in a model where low doses of poly(I∶C) are used as an adjuvant to induce a T cell response to an antigen [26], preferably a gluten antigen [27]. If the coordinated response of the innate and adaptive immune systems in such a model result in higher intestinal TG2 activity than can be achieved by either mechanism alone, then TG2 inhibition in such a model may result in biological consequences of relevance to celiac disease. Last but not least, the stepwise injection of three separate reagents (poly(I∶C), TG2 inhibitor, and 5BP) in our current mouse model is cumbersome, and could be improved. Usage of TG2 inhibitors that can be administered via oral gavage would not only simplify the protocol, but would also enable the evaluation of oral bioavailability of improved celiac drug candidates. At the same time, the simultaneous administration of “clickable” probes of activated TG2 in the mouse intestine would simplify both the in-life as well as post-sacrifice stages of our experimental protocol.

## Materials and Methods

### Antibodies

The antibodies used were anti-TG2 IgG produced in rabbit (Abcam, ab421) and Alexa fluor 488-conjugated goat anti-rabbit IgG (H-L) antibody (Invitrogen, A-11008). Alexa fluor 555-labeled streptavidin (Invitrogen, S-21381) was used to detect 5BP.

### Synthesis of TG2 inhibitors

Inhibitor **1** was synthesized as previously described [15].

### Poly(I∶C) preparation

Poly(I∶C) (Sigma, P1530) was dissolved in PBS at room temperature. The solution was heated to 85°C for 3 min, and subsequently annealed by allowing it to cool by 1°C per min, until it reached room temperature. The final poly(I∶C) concentration was measured at 260 nm, and used to inject mice at the desired concentration.

### Poly(I∶C) induced small intestinal injury in mice

C57BL/6 mice or TG2-knockout mice (Nanda et al., 2001) (8 weeks old) were IP injected with 30 mg/kg poly(I∶C) (Sigma, P1530) dissolved in sterile PBS to injure the small intestine as described previously [17]. Controls were given vehicle (PBS only). 5BP (Thermo scientific, 21345) was dissolved to 25 mg/ml in sterile PBS. Mice were IP injected twice with either 100 mg/kg 5BP or PBS at three hr intervals. For mice sacrificed after 9 hrs, 5BP was injected at 6 and 9 hrs. Mice were sacrificed using CO_2_ inhalation at the end of the experiment and blood samples were collected via cardiac puncture. The small intestines and lungs were collected and formalin-fixed for H&E staining or embedded in OCT and flash-frozen in liquid N2 for subsequent cryosectioning and immunohistochemistry.

### TG2 inhibition in mice

Compound **1** was purified by HPLC, and dissolved in 20% DMSO in sterile PBS at 4 mg/ml for intraperitoneal injections. Controls received vehicle only. Inhibitor was injected intra-peritoneally (IP) at 15 or 50 mg/kg.

### H&E staining

For histology, tissue from the small intestine was fixed in formalin and embedded in paraffin. Four-micrometer sections were affixed to slides, deparaffinized, and stained with hematoxylin and eosin (H&E) stain. Morphological changes in the stained sections were examined under light microscopy. Brightfield images were taken using a Zeiss Axiophot upright microscope with Axiovision software (version 4.2).

### Immunohistochemistry on frozen sections

Twenty µm cryosections were cut from OCT embedded tissues. Sections were fixed with 4% paraformaldehyde (PFA) for 10 min at RT. Sections were washed three times with PBS/0.1% Tween 20 (PBT) for 5 mins. Sections were then permeabilized with PBS/0.3% Triton X-100 for 10 min at RT, washed extensively with PBT two times for 5 mins, then blocked in 0.3% Triton X-100 and 5% goat serum in PBS (preblock buffer) in humid chamber as 300 µl per slide from 30 min – 4 hr at RT then probed with rabbit anti-transglutaminase 2 antibody (ab421) diluted in preblock buffer (1∶250) over night at 4°C. Slides were rinsed in PBS-Tween 20 (0.1% Tween-20) three times for 5 mins each then incubated with the Alexa-488 conjugated goat anti rabbit IgG (H+L) (A-11034, Invitrogen) (1∶250) and streptavidin-Alexa 555 diluted in preblock buffer (1∶250) for 45 mins at RT. Then, slides were washed three times in PBT for 5 mins each and stained with DAPI in PBT at RT for 5 mins and finally washed twice with PBT for 5 mins. Slides were mounted with 150 µl Mowiol 4–88 (Calbiochem) with 2.5% DABCO anti-photobleaching reagent (Sigma). Images were obtained on a Leica Sp2 confocal microscope, using LCS software.

### ELISA

Serum samples were kept frozen at −80°C until ready for cytokine measurement. The level of IL15R/IL-15 was measured using the mouse IL-15R/IL-15 complex ELISA Ready-SET-Go kit (eBioscience, 88-7215-22) from eBiosciences, following the manufacturer's protocol using serum at a 1∶20 dilution.
